# A Content Analysis of Arabic and English Newspapers before, during, and after the Human Papillomavirus Vaccination Campaign in the United Arab Emirates

**DOI:** 10.3389/fpubh.2016.00176

**Published:** 2016-08-29

**Authors:** Iffat Elbarazi, Hina Raheel, Kim Cummings, Tom Loney

**Affiliations:** ^1^Institute of Public Health, College of Medicine and Health Sciences, United Arab Emirates University, Al Ain, Abu Dhabi, United Arab Emirates; ^2^Department of Obstetrics and Gynaecology, College of Medicine and Health Sciences, United Arab Emirates University, Al Ain, Abu Dhabi, United Arab Emirates

**Keywords:** health communication, health promotion, human papillomavirus, human papillomavirus vaccine, United Arab Emirates, uterine cervical neoplasms, school health services

## Abstract

**Background:**

Cervical cancer is the fourth most common cancer among females in the United Arab Emirates (UAE) with an estimated incidence of 7.4 per 100,000 persons per year. In March 2008, the Health Authority of Abu Dhabi launched a free school-based campaign to provide all female Emirati students aged 15–17 years in the emirate of Abu Dhabi with the human papillomavirus vaccine (HPVV). Despite the proven efficacy of the HPVV in clinical trials, there has been limited research exploring the acceptance of this vaccine within a conservative Islamic society. The media plays a key role in changing beliefs and attitudes toward specific public health initiatives, such as vaccination programs. The primary aim of this study was to explore the content and communication style of the UAE newspapers (both Arabic and English) before, during, and after the HPV vaccination program.

**Methods:**

A systematic literature search was conducted on six national newspapers with the highest circulation figures in the UAE (Arabic: Al Ittihad, Al Khaleej, and Emarat El Youm; English: Khaleej Times, The National, and Gulf News) to retrieve articles related to cervical cancer prevention from January 2000 to May 2013. One bilingual researcher (Arabic–English) utilized content analysis to study the subject matter of communication in each article.

**Results:**

A total of 79 newspaper articles (*N* = 31 Arabic) were included in the study. Content analysis coding revealed five main themes: (i) “*HPV Screening or Vaccination Programmes in the UAE*” (*N* = 30); (ii) “*Cervical Cancer Statistics in the UAE*” (*N* = 22); (iii) “*Aetiology of Cervical Cancer and HPVV Efficacy*” (*N* = 12); (iv) “*Cultural Sensitivity and Misconceptions Surrounding HPVV in School-Aged Females*” (e.g., promoting promiscuity) (*N* = 8); and (v) “*Cost-Effectiveness, Efficacy, and Safety*” (*N* = 7).

**Conclusion:**

The UAE media is playing an important role in raising public awareness about cervical cancer and specific governmental health initiatives such as the HPVV program. Governmental health authorities may want to consider collaborating with the UAE media to develop a communication strategy to reduce the fears and misconceptions surrounding HPVV. Improved parental and adolescent knowledge on the HPVV may lead to increased acceptance and uptake in the UAE society.

## Introduction

Human papillomavirus (HPV) is a common sexually transmitted disease with ~40 different strains that can lead to genital warts and cervical, anal, penile, and vulvar cancers (1). Genotypes 16 and 18 of the virus cause ~70% of all cervical cancers and precancerous cervical lesions worldwide (1, 2). Cervical cancer is the fourth most common cancer in women, and the seventh overall, with an estimated 528,000 new cases and 266,000 deaths in 2012 (1, 3). Although the United Arab Emirates (UAE) has a relatively young national population structure, cancer is the third leading cause of mortality, and cervical cancer is the fourth most common cancer among females in the UAE with an estimated age-standardized incidence of 9.5 and mortality of 4.4 per 100 000 persons per year (4, 5). Approximately, half of all cases of cervical cancer occur in women between 35 and 55 years of age and 70% of cervical cancer cases are diagnosed at a late stage due to the lack of awareness on cervical cancer screening and the cost of screening in the UAE (6). As such, there is an urgent need to increase rates of cervical cancer prevention and early detection in the UAE.

In 2006, the United States (US) Food and Drug Administration approved the first preventive HPV vaccine (HPVV) Gardasil and in 2007 the US Advisory Committee on Immunization Practices recommended routine vaccination of females 11–12 years of age (7, 8). Previous studies have shown that the HPVV is safe and highly efficacious in reducing the risk of acquiring HPV infection of genotypes 16 and 18 (9–12). As such, a well-implemented HPVV program with good coverage and uptake is expected to be a more cost-effective measure to reduce the public health and economic burden of cervical cancer within a specific population compared with treatment and screening costs (11, 13). According to the WHO (1), 88% of HPV cases occur in low-income countries, largely due to the lack of regular screening (1, 3). However, in a high income country like the UAE, the major barrier to an effective cervical screening program is not economic but cultural as women are less likely to seek preventive internal examinations (14, 15). Therefore, vaccination campaigns in Islamic countries may be more effective than traditional screening programs in Westernized countries due to cultural barriers related to health education on sexually transmitted diseases and a reluctance to participate in a cervical screening program.

### Human Papillomavirus Virus Vaccination in the UAE

Abu Dhabi is the largest of the seven UAE emirates in terms of land mass and population [2012 mid-year estimates ≈2.3 million; (16)]. In March 2008, the Health Authority Abu Dhabi (HAAD) launched a campaign to vaccinate all girls in Abu Dhabi public schools in the first high-school years. The annual uptake rates of the Abu Dhabi school-based HPVV program ranged from 60 to 80% between 2007 and 2011 with higher uptake rates in public government schools compared with private schools (17). Many factors can affect the acceptability and hence uptake of the HPVV. These factors range from beliefs and perceptions of vaccine effectiveness and susceptibility, parental attitudes, sexual and cultural practices, provider attitudes, setting, and cost and availability of the vaccine (18). Vaccination against cervical cancer can be a controversial issue in many countries, as its opponents claim that it encourages promiscuity (19, 20).

A challenging obstacle that can affect the acceptance of the vaccine in UAE is that the vaccine targets a sexually transmitted disease that is still a taboo subject that can lead to stereotypes and stigmatization when this issue is discussed. The UAE is a conservative Muslim society, and numerous factors can play a role in deterring women and guardians from consenting for the vaccine. These factors include certain beliefs such as that the vaccine might encourage girls to become promiscuous and sexually active before marriage. As a Muslim country, it is illegal to commit adultery, and women are supposed to start their sexual activity after marriage; therefore, the UAE HPVV campaign targets females above the age of 15 years and also during their premarital processing tests. The UAE HPVV vaccination program has faced many challenges; among them, the acceptability of the vaccine amid parents, spouses, and providers due to its cultural sensitivity. Ortashi and colleagues conducted a series of studies to explore the knowledge, attitudes, and behaviors of these groups with regard to the HPVV (21–24). Ortashi et al. (21) conducted a cross-sectional study to assess the knowledge and attitude of school nurses (*n* = 125) about the HPVV in the emirate of Abu Dhabi, and 48% of participants reported cultural or religious unacceptability and 21% cited women’s lack of concern about their own health as major barriers toward 100% vaccination uptake in the UAE. Moreover, the majority (87%) of the nurses reported that they would not feel comfortable counseling high-school-aged females or parents about HPVV (21). Ortashi et al. (22) also conducted a questionnaire-based cross-sectional study to assess the knowledge and acceptability of the HPVV among male University students (*n* = 356) in the UAE. Less than one-third (32%) of male University students had ever heard of HPV and only 24% indicated that they would definitely accept the HPVV (22). The factors rated most likely to prevent students from using the vaccine were fear of the side effects (85%), absence of clear benefits (38%), and objections from a religious authority (25%). Interestingly, marital status and sexual activity were associated with greater knowledge of HPV but not with greater acceptance of vaccination (22). Finally, Ortashi and colleagues conducted a cross-sectional study utilizing a convenient sample of women (both UAE nationals and non-nationals) aged 18–50 years (*n* = 640) from the emirate of Abu Dhabi to assess their knowledge of HPV infection and HPVV in the UAE (23, 24). Less than one-third (29%) of the sample had ever heard of HPV infection, only 15% recognized it as a sexually transmitted infection, and less than one-quarter (22%) had heard of the HPVV (23, 24). Husband’s level of education was positively associated with better knowledge of HPV infection (23, 24). Overall, the knowledge of HPV infection and vaccine is low in the UAE.

Numerous researchers have suggested that designing effective vaccine-delivery strategies maximizes the potential of vaccines to decrease the heath and economic cancer burden of HPV (9, 11). A successful vaccination program will require the support of public health authorities, the coordination of health workers from different areas, and increased public awareness, acceptance, and uptake (9). Specifically, mass media communication is usually employed in health promotion to raise public health awareness, to create a climate of opinion conducive to policy change, and to shift attitudes and prompt behavior change through stressing the negative effects of health damaging behaviors and the benefits of health-conducive behaviors (25). Therefore, the media has the potential to reach and deliver targeted and tailored health messages based on current scientific evidence to large proportions of the population who may benefit from the HPVV. Several researchers have conducted content analysis of national newspapers articles in Canada and the US to examine the news information presented related to HPVV and concluded that the media, particularly newspapers, can be a powerful tool to promote vaccine uptake or to induce fear among the public against the use of HPVV (26–28). To the best of our knowledge, there have been no studies exploring the framing of public health messages related to cervical cancer and HPVV in the newspapers of an Islamic country such as the UAE. The primary aim of this study was to address this gap by analyzing the content and communication style of UAE newspaper coverage on cervical cancer and HPVV over a 13-year period from 2000 to 2013, before and after the HPV vaccination program was implemented in the emirate of Abu Dhabi.

## Materials and Methods

### Sample Selection and Data Sources

Arabic is the official language in the UAE, and English is also widely spoken due to the diversity of the expatriates living in the UAE. A census of all newspaper articles reporting on cervical cancer, HPV, and HPVV over a 13-year period (January 2000 to May 2013) was collected from six national newspapers (three Arabic and three English) with the highest circulation figures in the UAE (29). The print and online news outlets included in the study were *Al Ittihad, Al Khaleej, Emarat El Youm* (all Arabic), *Al Khaleej Times, Gulf News*, and *The National* (all English). These newspapers were selected based on their popularity and their distribution. According to the Arab Media Outlook Report 2011–2015 (29), *Gulf News* is the most popular and widely read newspaper in the UAE, followed by *Al Khaleej, Al Emarat Al Youm*, and *Khaleej Times*. The Arab Media Outlook Report conducted market research on the UAE population and 48% of the sample reported reading the *Gulf News*, 39% the *Al Khaleej*, 32% the *Al Emarat Al Youm*, 28% the *Khaleej* Times, and 27% the *Al Ittihad* newspaper (note: respondents reported reading multiple newspapers) (29). In terms of reported subscription rates, 34% of respondents reported subscribing to *Gulf News*, 17% to *Al Ittihad*, 14% *Khaleej Times*, and 12% the *Al Khaleej* newspaper.

### News Coverage Identification

The systematic literature search included searching the six UAE newspapers with the highest circulation figures for articles about cervical cancer screening and HPVV (29). Internet search engines and individual newspaper search engines were used to investigate the period from January 2000 to May 2013 employing a combination of MESH terms, free-text words, and entry terms “(HPV OR human papillomavirus) AND cervical cancer AND (Gardasil OR Cervarix OR vaccine).” This specific time period was before and after the implementation of the HPVV program in the emirate of Abu Dhabi. Only articles that discussed cervical cancer screening, prevention, and vaccination were included in the content analysis. Articles that discussed cancer in general including cervical cancer were excluded to avoid bias.

### Data Coding Process and Analysis

The unit of analysis was each newspaper article. Descriptive statistics were calculated to determine the proportion of news coverage in different newspapers and languages. Each article was examined to inquire about the frequency of general information about the HPV vaccine, the types of sources referenced in articles, and headlines and message frames coded using emergent themes. One bilingual researcher (Arabic–English) utilized content analysis to study the subject matter of communication in each article. Specifically, the researcher attempted to gain familiarity with the article by re-reading and reviewing them to gain a deeper understanding of the health message framing while bracketing their own preconceived beliefs about cervical cancer or HPVV. To determine the overall content and framing of the article, the researcher annotated the article text by attaching key words to segments of text. An “*emergent*” coding procedure was used in contrast to “*priori*” codes as this method allows the codes to reflect the views of the journalist rather than limiting the analysis to “*prefigured*” codes dictated by theory.

## Results

### Media Coverage by Language and Newspaper

A total of 79 articles were identified about the HPVV from 6 national newspapers in the UAE (Arabic: Al Ittihad, Al Khaleej, and Emarat El Youm; English: Al Khaleej, The National, and Gulf News) for the period from January 1, 2000 to June 30, 2013. Overall, 59.5% of articles were published in English newspapers, and 40.5% were published in Arabic newspapers. The distribution of articles in the English newspapers was 46.8% in *Khaleej Times*, 31.9% in *Gulf News*, and 21.3% in *The National* newspaper. In the Arabic newspapers, the distribution of the articles was 53.1% in *Al Emarat Elyoum*, 37.5% in *Al Ittihad*, and 9.4% in the *Al Khaleej* newspaper (Table 1).

**Table 1:** **Breakdown of newspaper articles by year and newspaper**.

Year	The national	Gulf news	Khaleej times	Al Iitihad	Al Khaleej	Al Emarat Elyoum	No. of articles
2001	0	1	0	0	0	0	1
2006	0	3	1	0	0	0	4
2007	0	3	4	0	0	0	7
**Launch of Abu Dhabi HPV vaccination campaign**							
2008	0	2	4	1	0	4	11
2009	0	2	3	0	0	4	9
2010	0	0	2	0	0	0	2
2011	3	1	3	1	0	3	11
2012	2	2	4	2	1	3	14
2013	4	1	2	8	2	3	20
Total	9	15	23	12	3	17	79

### Coverage by Month and Year

**Figure 1:**
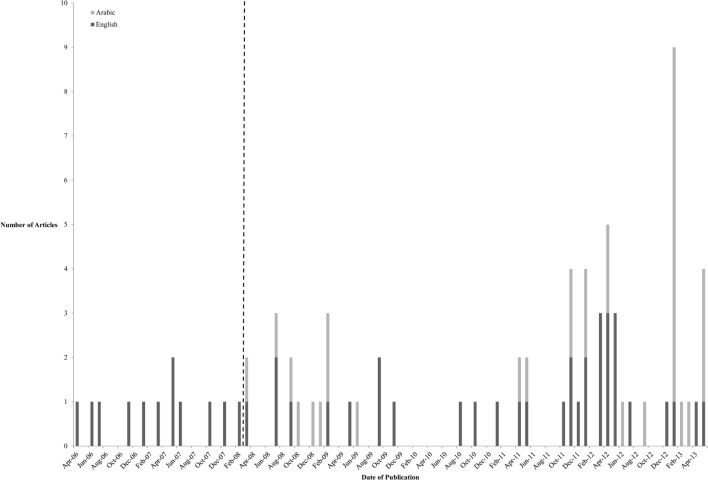
**Graph showing distribution of HPVV articles in Arabic and in English newspaper between April 2006 and May 2013**. Dotted line represents the launch of Health Authority Abu Dhabi’s HPV campaign in March 2008 to vaccinate all UAE National girls in Abu Dhabi public high schools in the first high-school years.

In the English newspapers, there was a fairly constant number of articles published about HPV, cervical cancer, or HPVV across the time period with small peaks in March (6.4%), April (6.4%), and May 2012 (6.4%; Table 1; Figure 1). However, there were distinct spikes in the number of articles published in the Arabic newspapers in January (25.0%) and June 2013 (9.4%).

### Article Frames and Themes

Five major article content themes were identified: (i) “*HPV Screening or Vaccination Programmes in the UAE*” (*N* = 30); (ii) “*Cervical Cancer Statistics in the UAE*” (*N* = 22); (iii) “*Aetiology of Cervical Cancer and HPVV Efficacy*” (*N* = 12); (iv) “*Cultural Sensitivity and Misconceptions Surrounding HPVV in School-Aged Females*” (e.g., promoting promiscuity) (*N* = 8); (v) “*Cost-Effectiveness, Safety, and Side Effects of HPVV*” (*N* = 7) (Table 2). Majority of articles (*N* = 30) discussed HAAD and other authorities’ campaigns and activities related to HPV screening and awareness programs (Table 2; Table S1 in Supplementary Material). Only eight articles discussed the cultural sensitivity and misconceptions issues related to HPVV in the UAE, and seven articles addressed the cost of the HPVV in general and the importance of including non-national citizens within the vaccination program (Table 2; Table S1 in Supplementary Material). Finally, 22 articles discussed cervical cancer statistics vaccination and screening programs, and 12 articles discussed the etiology of the vaccine and its efficacy (Table 2; Table S1 in Supplementary Material). Newspapers articles are listed in Table S1 in Supplementary Material. Overall, articles showed a general trend toward advocating for the vaccine and screening program in a non-direct manner by communicating data and statistics in an informative rather than persuasive style.

**Table 2:** **Number (%) of articles published related to each content theme**.

Theme	Number of articles (%)
HPV Screening or Vaccination Programmes in the UAE	30 (38)
Cervical Cancer Statistics in the UAE	22 (28)
Aetiology of Cervical Cancer and HPVV Efficacy	12 (15)
Cultural Sensitivity and Misconceptions Surrounding HPVV	8 (10)
Cost-Effectiveness, Safety, and Side Effects of HPVV	7 (9)

*HAAD, Health Authority Abu Dhabi*.

## Discussion

All selected Arabic and English newspapers highlighted the efforts of the government and local health authorities to reduce cervical cancer cases in the country and described the programs (e.g., screening and vaccination) implemented in the country to achieve their goal. According to Kelly et al. (30), media coverage around the cervical cancer vaccine increased significantly after the initial approval of the vaccine in 2006 in many countries. This trend was also observed in the UAE. Newspapers in the UAE started communicating data on the prevalence and incidence of cervical cancer globally and in the UAE from as early as 2006. The volume of articles increased significantly from 2008 the year of the launch of the vaccination program (Table 1; Figure 1). The distinct spikes in the number of articles published in the Arabic newspapers in January (25.0%) and June 2013 (9.4%) may potentially be explained by the announcement of the intention of health authorities in other Emirates, particularly Dubai, to introduce HPV immunization as part of the authority’s public health initiatives. However, the limited number of articles published in the past 10 years suggests that cervical cancer is perceived as an emerging public health issue in the UAE. Overall, the UAE newspaper articles provided superficial coverage of all of the issues surrounding the HPVV and focused on advertising and highlighting the cervical cancer-related activities of local UAE health authorities. The majority of articles described and discussed the burden of cervical cancer and the importance of screening and vaccination programs. For example, the UAE media discussed the need for vaccinations, the importance of reducing the number of women with cervical cancer, and the programs initiated by the authorities and policy makers to overcome this issue. However, the communication style of these articles was to inform the public about HPV and the vaccination program rather than to persuade the public, particularly parents, about the benefits of HPVV and overcome the misconceptions and stigma associated with the vaccine.

The academic literature is concerned with vaccine acceptance and strategies to change knowledge and attitudes toward any beneficial vaccine to maximize uptake. However, the newspaper articles reviewed in this study did not seem to focus on this important factor as a high priority. Rather, the media failed to discuss important matters such as strategies to increase the number of women completing timely pap smears and screening tests, encourage parents to vaccinate their daughters, and how to convince adolescents to receive the vaccination. This review of UAE newspapers mirrored the findings of that of Kelly et al. (30) and Penţa and Băban (31). Specifically, the vaccine efficacy and the Emirati societal need for the vaccine were highlighted using scientific evidence as there were recurrent references to the academic literature when the media discussed the efficacy of the vaccine and the impact of cervical cancer on the UAE society. A deeper discussion of HPVV acceptability and the burden of the disease and its effects on the UAE society were lacking. The English media highlighted the fact that non-nationals (expatriates constituting >85% of the UAE population) are not provided with the vaccine free of charge. The media content stressed the importance of introducing free vaccination for everyone to reduce the number of cases of cervical cancer and to improve the prevention program efficacy. In 2006, the Government of Abu Dhabi introduced Law No. 23 (of 20058) which outlined mandatory private health insurance for all expatriate residents. However, the range of services offered and treatments available for expatriate residents varies depending on their monthly salary, and the majority of health insurance schemes do not cover HPVV meaning expatriates would be required to pay out of pocket. Therefore, numerous newspaper articles highlighted the role of private insurance companies calling for full coverage for the vaccine and for cervical cancer screening tests by these companies to the entire UAE population included expatriates.

To modify the general public’s attitude toward a culturally sensitive topic in a conservative Islamic country such as the UAE, it is very important to use all available sources to reach the public. Mass media represented by printed and audio–visual means can reach a large proportion of the public especially parents and teachers who can disseminate the correct message and information. Printed and web-based newspaper media can be a useful vehicle for policy makers to improve people’s knowledge, positively influence their attitudes, and potentially enhance their health through the promotion of healthy lifestyle behaviors or engagement in specific public health programs such as vaccinations. In relation to HPVV, it is important to change people’s attitude and knowledge, especially when the issue can be culturally sensitive (30). The HPVV has been controversial as there are fears that it encourages teenagers to participate in pre-marriage sexual activities (19, 20) which are not culturally acceptable and illegal in the UAE. Our review of the UAE newspaper articles published around cervical cancer screening and vaccination showed that the discussion around socio-cultural issues related to HPVV in the UAE was superficial and shallow. It seems that the primary role of the UAE media was to provide information about the availability and efficacy of HPVV without discussing issues related to the acceptance and uptake of the vaccine, safety concerns, or cultural sensitivity. Kelly et al. (30) suggest that one factor contributing to the lack of knowledge about HPVV may be related to the lack of news coverage which may negatively impact people’s adoption of healthy behaviors like vaccination and screening.

Naidoo and Wills (25) list four different models on how the media can affect audiences and influence public health. The first model is the direct effect that manipulates the passive audience. The second model is the two-step model where mass communication influences key opinion leaders who are active members of the audience and who will spread ideas to other people through interpersonal communication. The third model suggests that people use the media to meet their needs by reinforcing existing beliefs or rejecting new ones. The last model is the cultural effects which indicate that media has a key role in creating beliefs and values about health, illnesses, and diseases. Our review revealed that Arabic and English newspapers in the UAE do not seem to follow any of these models as there is no obvious use for an approach to manipulate passive audience or to influence key opinion leaders. Also, the existing media do not reinforce existing beliefs or reject new ones and do not have much influence on beliefs and values illnesses and diseases. Upon reviewing the newspaper articles included in this study, it is clear that the power of mass communication is not well used in UAE context to promote HPVV uptake and to increase education and awareness. Although there were some timid efforts to discuss this topic, the media is required to be more proactive in terms of raising awareness and educating the public. The analysis of the media that discusses HPV, HPVV, and cervical cancer shows that there is still missing information on HPV prevention, transmission, symptoms, screening, and the importance of the vaccine. There is a dearth of discussion on the commonly held parental misconceptions (e.g., HPVV encourages promiscuous behavior) with the existing media preferring to concentrate on publicizing the local health authorities’ efforts, programs, and future plans.

One of the major strengths of this study is the inclusion of newspapers in two different languages to ensure a comprehensive coverage of newspaper articles as the UAE is a multicultural country with Arabic as the official language and English widely spoken. A weakness of the study was the exclusion of other forms of media (e.g., websites, radio, and television) and social media (e.g., twitter) that may have covered cervical cancer and HPVV and reached the younger segments of the UAE population. In addition, newspapers targeting specific nationalities within the UAE population (e.g., The Indian Times) were not included; rather, we focused on the Arabic and English national newspapers with the highest circulation and/or subscriptions rates that are most likely to be read by UAE nationals.

## Conclusion

Printed and web-based newspapers have the potential to act as a powerful persuader or discourager of public health behaviors in any country. Newspapers can play a positive role in changing people’s attitude toward HPVV and promoting vaccine acceptance by community groups, particularly middle-aged and older male adults who may be responsible for deciding whether or not a female family member receives the HPVV. The UAE media can potentially accelerate the acceptance and uptake of the vaccine which may help to reduce cervical cancer-related morbidity and mortality in the UAE. Governmental health authorities may want to consider collaborating with public health researchers, communication specialists, and the UAE media to develop a campaign strategy to reduce the fears and misconceptions surrounding HPVV.

## Author Contributions

IE conceived the idea. IE, HR, KC, and TL developed the protocol and conducted the literature search for newspaper articles. IE and HR reviewed the articles and conducted the thematic analysis. IE drafted the manuscript, and HR, KC, and TL revised the manuscript. All authors have critically reviewed, provided intellectual input to the manuscript, and approved the final version of the manuscript.

## Conflict of Interest Statement

The authors declare that the research was conducted in the absence of any commercial or financial relationships that could be construed as a potential conflict of interest.
