# Supplementary material for: A Content Analysis of Arabic and English Newspapers before, during, and after the Human Papillomavirus Vaccination Campaign in the United Arab Emirates
Source: Front Public Health. 2016 Aug 29;4:176. doi: 10.3389/fpubh.2016.00176 (PMC5002875; doi:10.3389/fpubh.2016.00176)
Supplement: Supplementary file 1 [file Table_1.docx]

**Supplementary File for Appendix**

**Table S1. Articles Included in Content Analysis from January 2000 to May 2013 by Newspaper**

| **Date** | **Paper** | **Language** | **Title** | **Topic** | **Section of Paper** | **Weblink** |
| --- | --- | --- | --- | --- | --- | --- |
| 06 Apr 2011 | **TN** | ENG | Vaccination Attack Cervical Cancer | Reporting on the vaccination program in schools/phases of the program and the uptake | Health | <http://www.thenational.ae/news/uae-news/health/vaccinations-attack-cervical-cancer> |
| 26 Nov 2011 | **TN** | ENG | Better cervical cancer screening is needed | Calling for a system that track down women to ensure regular they receive cervical cancer screening/ also reporting on the importance of the vaccine | Health | <http://www.thenational.ae/news/uae-news/health/better-cervical-cancer-screening-is-needed> |
| 22 Dec 2011 | **TN** | ENG | Cost puts women off crucial screening | **Highlighting** the cost problem increasing cervical cancer screening | Health | <http://www.thenational.ae/news/uae-news/health/cost-puts-women-off-crucial-screening> |
| 12 Jan 2012 | **TN** | ENG | Awareness campaign starts to fight cancer | Reporting on the nationwide campaign launched to raise awareness about cervical cancer | Health | <http://www.thenational.ae/news/uae-news/health/awareness-campaign-starts-to-fight-cancer> |
| 19 Mar 2012 | **TN** | ENG | Prevention for main cervical cancer cause | Reporting on a study that found out that 64 per cent of cervical cancer are caused by HPY type 16 and the importance of vaccination and screening | Health | <http://www.thenational.ae/news/uae-news/health/prevention-for-main-cervical-cancer-cause> |
| 12 Apr 2013 | **TN** | ENG | Health Authority Abu Dhabi introduces cervical cancer testing scheme | Reporting on the cervical cancer screening scheme that targets UAE women nationals , encouraging non -national to seek screening and to check their insurance/ also information on vaccine was presented | Health | <http://www.thenational.ae/news/uae-news/health/health-authority-abu-dhabi-introduces-cervical-cancer-testing-scheme> |
| 14 May 2013 | **TN** | ENG | Free vaccination against cervical cancer for Abu Dhabi women | Reporting on the nationwide immunization program | Health | <http://www.thenational.ae/news/uae-news/health/free-vaccination-against-cervical-cancer-for-abu-dhabi-women> |
| 20 May 2013 | **TN** | ENG | Cultural sensitivity can increase risk of cervical cancer | Addressing cultural issues and misconceptions regarding the screening in UAE/reporting also from authorities and from study done in Dubai | Health | <http://www.thenational.ae/thenationalconversation/comment/cultural-sensitivity-can-increase-risk-of-cervical-cancer> |
| 22 May 2013 | **TN** | ENG | Abu Dhabi 'cancer wave' initiative saves lives | Highlighting the achievement of the HAAD cancer awareness programs. Uptake of vaccine jumped 6% to reach 90% in schools from the period of OC 2012-March 2013 | Health | <http://www.thenational.ae/news/uae-news/health/abu-dhabi-cancer-wave-initiative-saves-lives> |
| 02 Jan 2001 | **GN** | ENG | Rising awareness | Reporting on an awareness program and the importance of early detection | UAE/ General | <http://gulfnews.com/news/gulf/uae/general/rising-awareness-1.407090> |
| 01 Apr 2006 | **GN** | ENG | Doctor: cervical cancer vaccinations vital | Reporting on the importance of the vaccine from a London medical conference | World/UK | <http://gulfnews.com/news/world/uk/doctor-cervical-cancer-vaccinations-vital-1.231239> |
| 01 Jul 2006 | **GN** | ENG | Cervical cancer shots recommneded for girls | Recommendation for Gardasil shots in Atlanta | World/other world stories | <http://gulfnews.com/news/world/other-world/cervical-cancer-shots-recommended-for-girls-1.242992> |
| 25 Nov 2006 | **GN** | ENG | Cervical cancer vaccine in immunisation plan sought | Discussing the controversy of the vaccination plan in UAE and the | UAE/Health | <http://gulfnews.com/news/gulf/uae/health/cervical-cancer-vaccine-in-immunisation-plan-sought-1.266889> |
| 08 May 2007 | **GN** | ENG | Sharjah residents to be screened for cancer | Reporting on Sharjah program of screening | UAE/Health | <http://gulfnews.com/news/gulf/uae/health/sharjah-residents-to-be-screened-for-cancer-1.177600> |
| 15 Dec 2007 | **GN** | ENG | A question of age for cervical cancer vaccination in UAE | Reporting on the concerns around the vaccine and stressing that age is the stumbling block in vaccinating girls in UAE and not that it encourages promiscuity | UAE/Health | <http://gulfnews.com/news/gulf/uae/health/a-question-of-age-for-cervical-cancer-vaccination-in-uae-1.218236> |
| 27 Oct 2007 | **GN** | ENG | Girls aged 12 to 13 to get cervical cancer jabs | Reporting on the vaccination plan in UK | World/UK | <http://gulfnews.com/news/world/uk/girls-aged-12-to-13-to-get-cervical-cancer-jabs-1.208091> |
| 04 Feb 2008 | **GN** | ENG | Abu Dhabi to vaccinate girls against cancer | Highlighting the sensitivity of the vaccine and the controversy it creates | UAE/Health | <http://gulfnews.com/news/gulf/uae/health/abu-dhabi-to-vaccinate-girls-against-cancer-1.83147> |
| 08 Jul 2008 | **GN** | ENG | Dubai plans cervical cancer vaccine for women | Discussing the plans of vaccination | UAE/Health | <http://gulfnews.com/news/gulf/uae/health/dubai-plans-cervical-cancer-vaccine-for-women-1.117131> |
| 29 Sep 2009 | **GN** | ENG | British girl dies after cervical cancer vaccine | Reporting on the death of a girl in UK because of the vaccine (Cervarix)and the controversy of the vaccine and whether there will be a plan | World /UK | <http://gulfnews.com/news/world/uk/british-girl-dies-after-cervical-cancer-vaccine-1.541244> |
| 15 May 2009 | **GN** | ENG | Promoting cervical cancer awareness | Raising the alarm about the impact of cervical cancer and the importance of screening | Opinions/ Editorials | <http://gulfnews.com/opinions/editorials/promoting-cervical-cancer-awareness-1.68757> |
| 31 Oct 2011 | **GN** | ENG | Vaccine boosts fight against cervical cancer | Reporting on the availability of the vaccine by health authorities and the importance of education about cervical cancer. The article provide statistics | UAE/Government | <http://gulfnews.com/news/gulf/uae/government/vaccine-boosts-fight-against-cervical-cancer-1.920937> |
| 15 Apr 2012 | **GN** | ENG | Appeal to make vaccination mandatory | Recommending premarriage vaccination in UAE by Hamadan Award | UAE/Health | <http://gulfnews.com/news/gulf/uae/health/appeal-to-make-cancer-vaccination-mandatory-1.1008398> |
| 27 Jul 2012 | **GN** | ENG | Time is running out to get vaccinated | Reporting on the importance of vaccination | Health | <http://m.gulfnews.com/news/uae/health/time-running-out-to-get-vaccinated-1.1054186> |
| 24 Apr 2013 | **GN** | ENG | Cervical cancer vaccination could be mandatory in UAE | Reporting on the vaccination program and schedule in UAE from Dubai Health Authority and reporting from an event officials that marked world immunisation week | Health | <http://m.gulfnews.com/news/uae/health/cervical-cancer-vaccination-could-be-mandatory-in-uae-1.1174754> |
| 14 Jun 2006 | **KT** | ENG | New vaccine to prevent cervical cancer | Reporting on the availability of the vaccine and that it needs approval from MOH to introduce it in UAE | UAE | <http://www.khaleejtimes.com/kt-article-display-1.asp?section=theuae&xfile=data/theuae/2006/june/theuae_june400.xml> |
| 07 Jan 2007 | **KT** | ENG | UAE gets cervical cancer vaccine | Reporting on the availability of HPVV in UAE being the first in the Middle East to introduce it | UAE | <http://www.khaleejtimes.com/kt-article-display-1.asp?section=theuae&xfile=data/theuae/2007/january/theuae_january169.xml> |
| 06 Mar 2007 | **KT** | ENG | Cervical cancer on the rise in country | Giving statistics and reporting on an education program organized by American hospital of Dubai to raise awareness about HPVV | UAE | <http://www.khaleejtimes.com/kt-article-display-1.asp?section=theuae&xfile=data/theuae/2007/march/theuae_march160.xml> |
| 06 May 2007 | **KT** | ENG | Cervical cancer focus of Women's 3-F show | Reporting on a function that addressed 500 women and promoted the HPVV and highlighted the burden of cervical cancer on society | UAE | <http://www.khaleejtimes.com/kt-article-display-1.asp?section=theuae&xfile=data/theuae/2007/may/theuae_may148.xml> |
| 19 Jun 2007 | **KT** | ENG | Focus on cervical cancer | Reporting from HAAD some statistics and importance of vaccination that were discussed in a one day workshop organized by HAAD) | uae | <http://www.khaleejtimes.com/kt-article-display-1.asp?section=theuae&xfile=data/theuae/2007/june/theuae_june499.xml> |
| 09 Mar 2008 | **KT** | ENG | Vaccination: some parents against daughters taking it | Providing a short view on some parents about the vaccine and why they are refusing it | UAE | <http://www.khaleejtimes.com/kt-article-display-1.asp?section=theuae&xfile=data/theuae/2008/march/theuae_march251.xml> |
| 07 Jul 2008 | **KT** | ENG | Cervical cancer awareness drive launched in UAE | Reporting on the new launch of cervical cancer awareness programmes | UAE | <http://www.khaleejtimes.com/kt-article-display-1.asp?section=theuae&xfile=data/theuae/2008/july/theuae_july257.xml> |
| 12 Jul 2008 | **KT** | ENG | Osteoporosis and cervical cancer programmes soon | Reporting on the new launch of cervical cancer awareness programmes | UAE | <http://www.khaleejtimes.com/kt-article-display-1.asp?section=theuae&xfile=data/theuae/2008/july/theuae_july425.xml> |
| 22 Sep 2008 | **KT** | ENG | Cervical cancer test poised to be a boon in developing world | Reporting on the importance of screening. Some statistics are provide and a report on a study published by The Lancet Oncology on a promising new laboratory test that can detect HPV in the same visit and called care HPV | Paris | <http://www.khaleejtimes.com/kt-article-display-1.asp?section=health&xfile=data/health/2008/september/health_september27.xml> |
| 04 Feb 2009 | **KT** | ENG | Now, Toll Free Information on Cervical Cancer | Informing about the toll free number available for cervical cancer information | UAE | <http://www.khaleejtimes.com/kt-article-display-1.asp?section=theuae&xfile=data/theuae/2009/february/theuae_february87.xml> |
| 20 Sep 2009 | **KT** | ENG | FDA panel backs Glaxo’s cervical vaccine for women | Reporting on the new HPVV by Merck and the FDA approval of both vaccines by Merck (Cervarix) and by Glaxo smithKline (Gardasil) | USA | <http://www.khaleejtimes.com/kt-article-display-1.asp?section=health&xfile=data/health/2009/september/health_september26.xml> |
| 20 Nov 2009 | **KT** | ENG | New cervical cancer screening guidelines issued | Reporting on the new cervical cancer screening guidelines | USA | <http://www.khaleejtimes.com/kt-article-display-1.asp?section=health&xfile=data/health/2009/november/health_november59.xml> |
| 21 Aug 2010 | **KT** | ENG | Regular clinical screening helps prevent cervical cancer | Providing statistics and information on HPV and reinforcing the prevention and early screening | UAE | <http://www.khaleejtimes.com/kt-article-display-1.asp?section=theuae&xfile=data/theuae/2010/august/theuae_august568.xml> |
| 18 Oct 2010 | **KT** | ENG | Eight virus types cause almost all cervical cancer | Reporting on a study published by Lancet on types of HPV and reinforcing the importance of screening and vaccination | London | <http://www.khaleejtimes.com/kt-article-display-1.asp?section=health&xfile=data/health/2010/october/health_october38.xml> |
| 08 Jan 2011 | **KT** | ENG | Circumcision helps stop wart virus, study finds | Reporting from Reuters on a new study on circumcision and HPV infection from a study published in Lancet |  | <http://www.khaleejtimes.com/kt-article-display-1.asp?section=theuae&xfile=data/theuae/2011/january/theuae_january172.xml> |
| 20 May 2011 | **KT** | ENG | Concern voiced over plan to introduce HPV vaccine | Reporting on cultural issues concerning introduction of HPVV in Bahrain | Bahrain | <http://www.khaleejtimes.com/kt-article-display-1.asp?section=middleeast&xfile=data/middleeast/2011/may/middleeast_may503.xml> |
| 03 Nov 2011 | **KT** | ENG | Nationwide screening for cervical cancer urged | Reporting alarming statistics on cervical cancer and the importance of screening and prevention | UAE | <http://www.khaleejtimes.com/kt-article-display-1.asp?section=theuae&xfile=data/theuae/2011/november/theuae_november110.xml> |
| 11 Jan 2012 | **KT** | ENG | Cut in cervical cancer screening fee by 50% | Reporting on the cost of HPVV and the efforts to reduce of cost for screening and vaccination | UAE | <http://www.khaleejtimes.com/kt-article-display-1.asp?section=theuae&xfile=data/theuae/2012/january/theuae_january272.xml> |
| 12 Mar 2012 | **KT** | ENG | New test to prevent cervical cancer urged | Encouraging women to ask for HPV test while having a Pap test and Promoting screening | UAE | <http://www.khaleejtimes.com/kt-article-display-1.asp?section=theuae&xfile=data/theuae/2012/march/theuae_march356.xml> |
| 26 Mar 2012 | **KT** | ENG | Researcher for compulsory vaccination against HPV | Reporting on the views of 2 researchers from UAE University on making HPVV compulsory before marriage based on alarming statistics | UAE | <http://www.khaleejtimes.com/kt-article-display-1.asp?section=theuae&xfile=data/theuae/2012/march/theuae_march749.xml> |
| 22 Dec 2012 | **KT** | ENG | Prevention of cervical cancer | Tips on healthy lifestyle to prevent cervical cancer | UAE | <http://www.khaleejtimes.com/kt-article-display-1.asp?section=health&xfile=data/health/2012/december/health_december37.xml> |
| 08 Jan 2013 | **KT** | ENG | Cervical cancer cases on the rise in UAE | Reporting on statistics of cervical cancer and the prevention program by HAAD | UAE | <http://www.khaleejtimes.com/kt-article-display-1.asp?section=health&xfile=data/health/2013/january/health_january10.xml> |
| 14 May 2013 | **KT** | ENG | Get vaccinated early to avoid cervical cancer, say experts | Reporting on the vaccination program, HPV infection causes , statistics of cervical cancer and treatment success and the importance of getting the vaccination and screening | UAE | <http://www.khaleejtimes.com/kt-article-display-1.asp?section=health&xfile=data/health/2013/may/health_may18.xml> |
| 09 Dec 2008 | **AI** | ARA | German skepticism effectiveness of cervical cancer vaccine | Reporting German researchers’ criticism of the cervical cancer vaccine. German researchers do not believe that the vaccine is cost effective and that research results so far are contradictory | World | <http://www.alittihad.ae/details.php?id=50366&y=2008> |
| 10 May 2011 | **AI** | ARA | Azza Al Qasimi attend annual party of Sharjah Ladies club | Reporting on a campaign about cervical cancer danger | UAE | <http://www.alittihad.ae/details.php?id=44515&y=2011> |
| 17 Apr 2012 | **AI** | ARA | A campaign in the college of Medicine in UAE University about cancer disease | Reporting on a campaign to raise awareness among students in UAE university in the college of medicine | UAE | <http://www.alittihad.ae/details.php?id=379728&y-2012> |
| 12 Jun 2012 | **AI** | ARA | Discovery of cells responsible about cervical cancer | Reproting on a study from Harvard university | World | <http://www.alittihad.ae/details.php?id=57471y=2012> |
| 05 Jan 2013 | **AI** | ARA | Health Authority Abu Dhabi launches a campaign against cervical cancer | Reporting on the campaign launched to raise awareness about early detection and about the importance of the vaccine | UAE | <http://www.alittihad.ae/details.php?id=1635&y=2013> |
| 08 Jan 2013 | **AI** | ARA | 61 cases of cervical cancer in Abu Dhabi last year | Reporting on screening initiative | UAE | <http://www.alittihad.ae/details.php?id=2822&y=2013> |
| 12 Jan 2013 | **AI** | ARA | One test can detect three types of cancer | Reporting on a test that detect cervical, uterine and ovarian cancer | World | <http://www.alittihad.ae/details.php?id=4094&y=2013> |
| 12 Jan 2013 | **AI** | ARA | Health promotion and vaccination are the best way to prevent cervical cancer | Reporting on an activity at Zayed University and some statistics | UAE | <http://www.alittihad.ae/details.php?id=4111&y=2013> |
| 25 Jan 2013 | **AI** | ARA | Alain Police organises a lecture about cervical cancer | Reporting on the activity to promote awareness about cervical cancer in Abu Dhabi police | UAE | <http://www.alittihad.ae/details.php?id=9139&y=2013> |
| 07 Mar 2013 | **AI** | ARA | Abu Dhabi Police medical section organizes a series of lectures to educate women about prevention from cervical cancer | Reporting on the lectures and campaigns | UAE | <http://www.alittihad.ae/details.php?id=23244&y2013> |
| 13 May 2013 | **AI** | ARA | Free Cervical cancer Vaccination for UAEU students | Reporting on a health awareness campaign that offered HPV vaccine to UAEU female students | UAE | <http://www.alittihad.ae/details.php?id=46678&y=2013> |
| 14 May 2013 | **AI** | ARA | Health Authority Abu Dhabi launches a cervical cancer vaccine program | Reporting on the launch of the vaccination program for women between 18-26 | UAE | <http://www.alittihad.ae/details.php?id=47056&y=2013> |
| 12 Jan 2012 | **AK** | ARA | Launching of a campaign about prevention from cervical cancer | Statistics and reporting on the campaign | UAE | <http://www.alkhaleej.ae/portal/9e68a6fa-9739-443e-9686-fb7acf9449dd.aspx> |
| 08 Jan 2013 | **AK** | ARA | 61 cases of cervical cancer in AbuDhabi last year | Reporting on statistics | UAE | <http://www.alkhaleej.ae/portal/f1a8376b-d522-4de4-94dd-28dc3726c72e.aspx> |
| 25 Jan 2013 | **AK** | ARA | Abu Dhabi Police medical section organizes a series of lectures to educate women about prevention from cervical cancer | Reporting on the lectures and campaigns | UAE | <http://www.alkhaleej.ae/portal/b9cf2aa5-d4f8-4ba0-a2c3-5ac58dae854f.aspx> |
| 07 Mar 2008 | **AEE** | ARA | Parents are refusing to vaccinate their daughters against cervical cancer | Reporting parents refusal to vaccinate their girls as they believe that it promotes promiscuity and it is an insult to them to vaccinate their daughters against cervical cancer | UAE | <http://www.emaratalyoum.com/local-section/2008-03-07-1.185919> |
| 26 Oct 2008 | **AEE** | ARA | Big Efforts to vaccinate women with vaccine against cervical cancer | Reporting on the effectiveness of the vaccine Gardasil | World | <http://www.emaratalyoum.com/local-section/health/2013-05-14-1.574585> |
| 12 Jul 2008 | **AEE** | ARA | Side effects of a vaccine against cervical cancer | Reporting on side effects of vaccine in the US | World | <http://www.emaratalyoum.com/local-section/2008-07-12-1.171970> |
| 01 Sep 2008 | **AEE** | ARA | Coffee decrease risk of cervical cancer | Reporting on a study from Japan | World | <http://www.emaratalyoum.com/local-section/2008-09-01-1.233231> |
| 01 Jan 2009 | **AEE** | ARA | Traffic department and Health Authority against cervical cancer | Reporting on the cooperation between Road traffic department and health authority in the campaign against cervical cnacer | UAE | <http://www.emaratalyoum.com/local-section/2009-01-01-1.120495> |
| 03 Feb 2009 | **AEE** | ARA | A free number to increase awareness about cervical cancer | The UAE campaign has assigned a free number to educate women about cervical cancer | UAE | <http://www.emaratalyoum.com/local-section/2009-02-03-1.122743> |
| 08 Apr 2011 | **AEE** | ARA | Cervical cancer is threatening the death of 250 women in the next 5 years in the country | Reporting on statistics and rising the alarm | UAE | <http://www.emaratalyoum.com/local-section/health/2011-04-08-1.378521-> |
| 01 Nov 2011 | **AEE** | ARA | 55 new cases of cervical cancer cases in the country annually | Reporting on statistics | UAE | <http://www.emaratalyoum.com/local-section/health/2011-11-01-1.434429> |
| 10 Nov 2011 | **AEE** | ARA | Cervical cancer kills a woman every 2 minutes | Reporting on statistics | UAE | <http://www.emaratalyoum.com/life/life-style/2011-11-10-1.435984> |
| 10 Nov 2011 | **AEE** | ARA | Abu Dhabi Health Authority launches a campaign” Think about your health” | Reporting on the campaign that includes raising awareness about cervical cancer | UAE | <http://www.emaratalyoum.com/life/life-style/2011-11-10-1.435984> |
| 28 Jan 2012 | **AEE** | ARA | Abu Dhabi Health Authority appoints 10 centres to prevent cervical cancer | Reporting on the centres in Abu Dhabi that will conduct cervical cancer screening/Also the centres will contact the women to remind them of their testing by phone and electronically also the article reports some statistics | UAE | <http://www.emaratalyoum.com/local-section/health/2012-01-28-1.456272> |
| 13 Apr 2012 | **AEE** | ARA | Educating inmates in Abu Dhabi about cervical cancer danger | Reporting on program | UAE | <http://www.emaratalyoum.com/local-section/health/2012-04-13-1.476126> |
| 14 Sep 2012 | **AEE** | ARA | Misconceptions are preventing cervical cancer vaccination in private schools | Reporting on the misconceptions that are behind parents refusal of vaccinating their girls in private schools. Also reporting on statistics and programs | UAE | <http://www.emaratalyoum.com/local-section/health/2012-09-14-1.511591> |
| 08 Jan 2013 | **AEE** | ARA | Abu Dhabi Health Authority starts early screening for cervical cancer in 20 centres | Reporting on screening program | UAE | <http://www.emaratalyoum.com/local-section/health/2013-01-08-1.539483> |
| 25 Jan 2013 | **AEE** | ARA | A lecture about cervical cancer | Reporting on a lecture in Al Ain police health section | UAE | <http://www.emaratalyoum.com/local-section/other/2013-01-25-1.544086> |
| 09 Feb 2013 | **AEE** | ARA | Dubai Municipality centre educate attendees about cervical cancer prevention | Reporting on program | UAE | <http://www.emaratalyoum.com/local-section/health/2013-02-09-1.548188> |
| 14 May 2013 | **AEE** | ARA | Abu Dhabi Health Authority elevates the age of vaccination against cervical cancer to 26 years | Reporting on the vaccination program and the launch of the vaccine amongst women from 19-26 years | UAE | <http://www.emaratalyoum.com/local-section/health/2013-05-14-1.574585> |

*Note*. TN denotes The National; GN denotes Gulf News; KT denotes Khaleej Times; AI denotes Al Itihad; AK denotes Al Khaleej; AEE denotes Al Emarat Elyoum; ENG denotes English; ARA denotes Arabic.
